# Collagen-Binding Hepatocyte Growth Factor (HGF) alone or with a Gelatin- furfurylamine Hydrogel Enhances Functional Recovery in Mice after Spinal Cord Injury

**DOI:** 10.1038/s41598-018-19316-y

**Published:** 2018-01-17

**Authors:** Kentaro Yamane, Tetsuro Mazaki, Yasuyuki Shiozaki, Aki Yoshida, Kensuke Shinohara, Mariko Nakamura, Yasuhiro Yoshida, Di Zhou, Takashi Kitajima, Masato Tanaka, Yoshihiro Ito, Toshifumi Ozaki, Akihiro Matsukawa

**Affiliations:** 1 Department of Orthopaedic Surgery, Okayama University, 2-5-1 Shikata, Kita-ku, Okayama, 700-8558 Japan; 2 Department of Pathology and Experimental Medicine, Graduate School of Medicine, Dentistry and Pharmaceutical Sciences, Okayama University, 2-5-1 Shikata, Kita-ku, Okayama, 700-8558 Japan; 3 Speech-language Pathology Department, Kyushu University of Health and Welfare, 1714-1 Yoshino-cho, Nobeoka, Miyazaki, 882-8508 Japan; 4 Department of Biomaterials and Bioengineering, Faculty of Dental Medicine and Graduate School of Dental Medicine, Hokkaido University, Kita 13, Nishi 7, Kita-ku, Sapporo, 060-8586 Japan; 5 Nano Medical Engineering Laboratory, RIKEN, 2-1 Hirosawa, Wako, Saitama, 351-0198 Japan

## Abstract

The treatment of spinal cord injury (SCI) is currently a significant challenge. Hepatocyte growth factor (HGF) is a multipotent neurotrophic and neuroregenerative factor that can be beneficial for the treatment of SCI. However, immobilized HGF targeted to extracellular matrix may be more effective than diffusible, unmodified HGF. In this study, we evaluated the neurorestorative effects of an engineered HGF with a collagen biding domain (CBD-HGF). CBD-HGF remained in the spinal cord for 7 days after a single administration, while unmodified HGF was barely seen at 1 day. When a gelatin-furfurylamine (FA) hydrogel was applied on damaged spinal cord as a scaffold, CBD-HGF was retained in gelatin-FA hydrogel for 7 days, whereas HGF had faded by 1 day. A single administration of CBD-HGF enhanced recovery from spinal cord compression injury compared with HGF, as determined by motor recovery, and electrophysiological and immunohistochemical analyses. CBD-HGF alone failed to improve recovery from a complete transection injury, however CBD-HGF combined with gelatin-FA hydrogel promoted endogenous repair and recovery more effectively than HGF with hydrogel. These results suggest that engineered CBD-HGF has superior therapeutic effects than naïve HGF. CBD-HGF combined with hydrogel scaffold may be promising for the treatment of serious SCI.

## Introduction

Spinal cord injury (SCI), often caused by physical trauma, is an insult to the spinal cord resulting in a physically and psychologically devastating clinical condition with irreversible neurological deficits and disabilities^1^. SCI occurs worldwide, with an annual incidence of 10 to 83 cases per million and typically impacts younger individuals, reducing the quality of life and with estimated lifetime costs of more than US $4 million per person^2^. New advances from preclinical and clinical studies offer potential neuroprotective and therapeutic possibilities^3,4^; however, due to the poor intrinsic regenerative capability of the spinal cord, treatment of SCI is still a significant problem. A major challenge in repairing the injured spinal cord is how to regenerate tissue and encourage regrowth of severed axons with cellular and molecular therapies^5,6^, although several growth factors have been applied for the treatment of SCI^6^.

Hepatocyte growth factor (HGF), initially identified as a mitogenic factor for primary hepatocytes, is a multipotent neurotrophic and neuroregenerative factor with a wide variety of biological functions in the nervous system. The major role of HGF is to potentiate the response of developing neurons to specific signals^7^. HGF not only promotes neurogenesis and oligogliogenesis by neural stem cells, it exhibits anti-apoptotic functions for neurons and oligodendrocytes and inhibits glial scar formation^7–11^. HGF promotes endogenous repair and functional recovery after SCI in rats^10^ and marmosets^12^. Thus, HGF appears to be a viable approach for the treatment of SCI.

HGF can be delivered by direct injection into the injury site^10,12^ and immobilised HGF can be localized and retained at the targeted site for longer periods, thus extending the functional half-life of this factor. We previously created a novel HGF fused to an additional collagen-binding domain (CBD) derived from fibronectin (CBD-HGF), which binds to collagen matrices^13^. As a tissue-engineering approach, scaffolds have been attracting increasing attention^14,15^ as they provide an environment for cell attachment, proliferation and differentiation. Furthermore, scaffolds can be used to achieve drug delivery with high loading efficiency at specific sites. Hydrogels are leading candidates for engineered tissue scaffolds due to their unique compositional and structural similarities to the natural extracellular matrix^16^. In addition, hydrogels are suitable biomaterials for drug and cell delivery^16,17^. We recently developed a gelatin derivative modified with furfurylamine (gelatin-FA), which is rapidly consolidated by visible light in the presence of Rose Bengal (RB)^18^. Gelatin-FA effectively delivered collagen-binding bone morphogenetic protein-4 (CBD-BMP4) not only cells but also to affected sites, enhancing osteogenesis^18^.

In the present study, we applied our tissue engineering strategies to SCI. We demonstrated that CBD-HGF enhanced recovery from spinal cord compression injury compared with HGF. CBD-HGF combined with gelatin-FA hydrogel successfully promoted neural regeneration from a severe complete injury. Thus, a tissue engineering strategy composed of collagen-binding HGF and a gelatin scaffold is promising for the treatment of SCI.

## Results

### Binding capacity of CBD-HGF to spinal cord tissue

**Figure 1:**
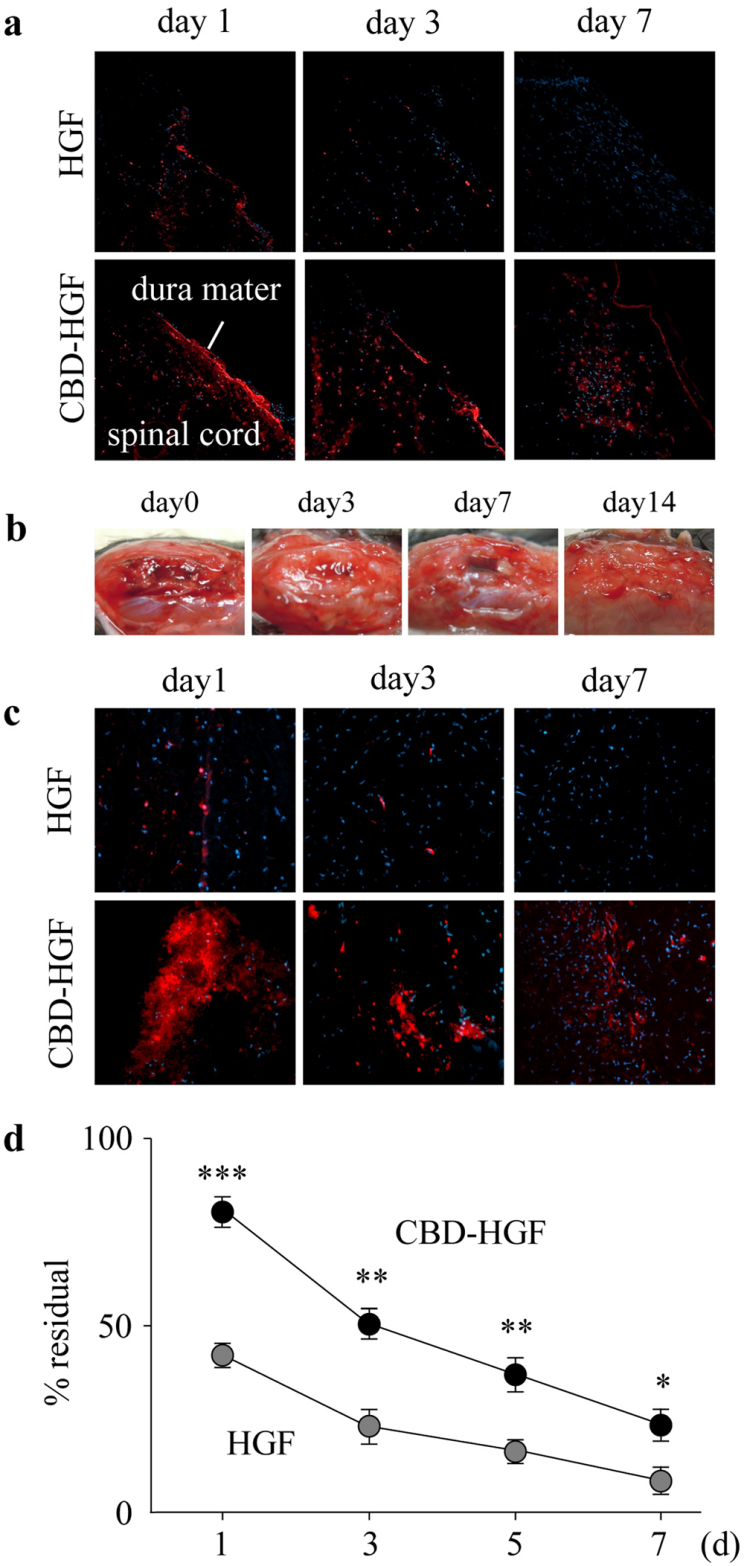
Binding capacity of CBD-HGF. Fluorescence-labelled CBD-HGF and HGF were administered into spinal cords (**a**) and gelatin-FA hydrogels were implanted into injured dura mater (**b**,**c**). At appropriate time intervals after the procedure, mice were killed and the sections were examined by fluorescence microscopy. (**a**,**c**) HiLyte Fluor 555-labelled CBD-HGF/HGF is shown in red. Representative photographs from each group (3 mice) are shown. (**b**) Representative gross appearance after the procedure (3 mice). (**d**) Gelatin-FA solution containing CBD-HGF or HGF (each 100 ng) was gelled by irradiation with visible light and incubated with PBS at 37 °C. Concentrations of HGF in the remaining gel were measured by ELISA (n = 4 each). *p < 0.05, **p < 0.01, ***p < 0.001.

We first asked whether CBD-HGF could enhance the tissue-binding capacity of HGF to spinal cord tissue. Fluorescently labelled native HGF or CBD-HGF were administered into spinal cords and examined using fluorescence microscopy after 1 and 7 days. Although HGF was barely seen on day 1, CBD-HGF showed a strong signal that remained until day 7 (Fig. 1a). Thus, CBD-HGF had a higher binding affinity to spinal cord than native HGF.

Next, we placed gelatin solutions (15% gelatin-FA and 0.05% RB) containing fluorescently labelled HGF or CBD-HGF onto spinal cords and photo-cross-linked them with visible light. Macroscopically, the gelled gelatin-FA remained for 7 days after implantation, and the implantation site was indistinguishable from the surrounding tissue on day 14 (Fig. 1b). Microscopically, HGF had diffused and faded by day 1, while a much stronger CBD-HGF signal was seen on day 1 relative to HGF and the signal was present at day 7 (Fig. 1c). To further confirm the binding capacity of CBD-HGF to gelatin-FA hydrogel, HGF and CBD-HGF were suspended in gelatin solutions, consolidated by visible light and then incubated in PBS. As shown in Fig. 1d, a higher level of CBD-HGF remained in gelatin-FA at each time point compared with HGF. Thus, gelatin-FA appeared to be a suitable scaffold for HGF-based tissue engineering.

### Treatment of spinal cord compression injury by CBD-HGF

**Figure 2:**
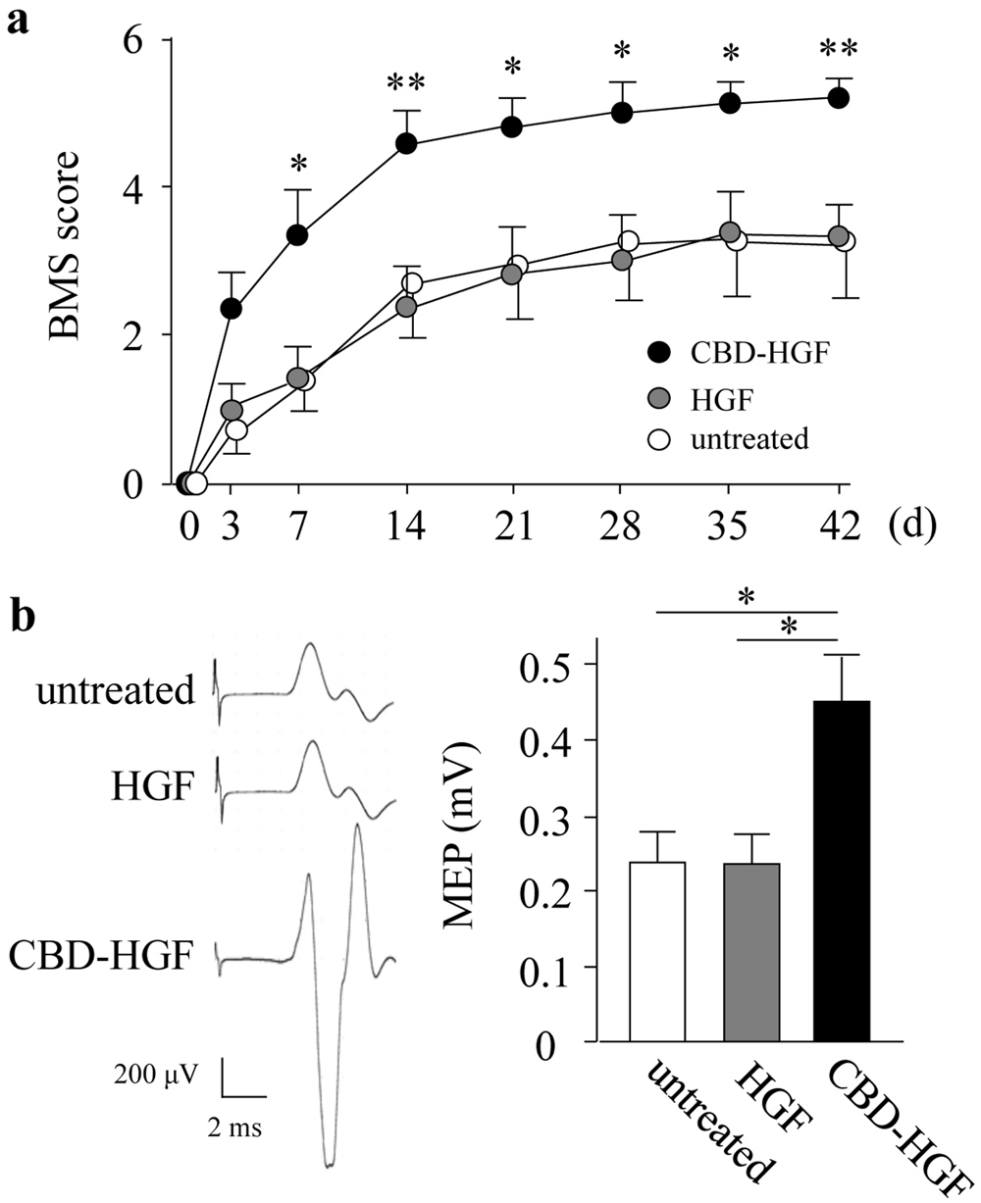
Treatment of spinal cord compression injury by CBD-HGF. Mice subjected to a compression injury were treated with a single administration of CBD-HGF or HGF. Untreated mice were used as controls. (**a**) Motor function recovery was assessed at 6 weeks after treatment using the BMS score (8 mice each per 3 groups). *p < 0.05, **p < 0.01 versus untreated control. (**b**) MEP analysis was performed as an electrophysiological assessment at 6 weeks after treatment (6 mice each per 3 groups). Left: Representative data from each group. Right: Quantitative data from the 3 groups; *p < 0.05.

**Figure 3:**
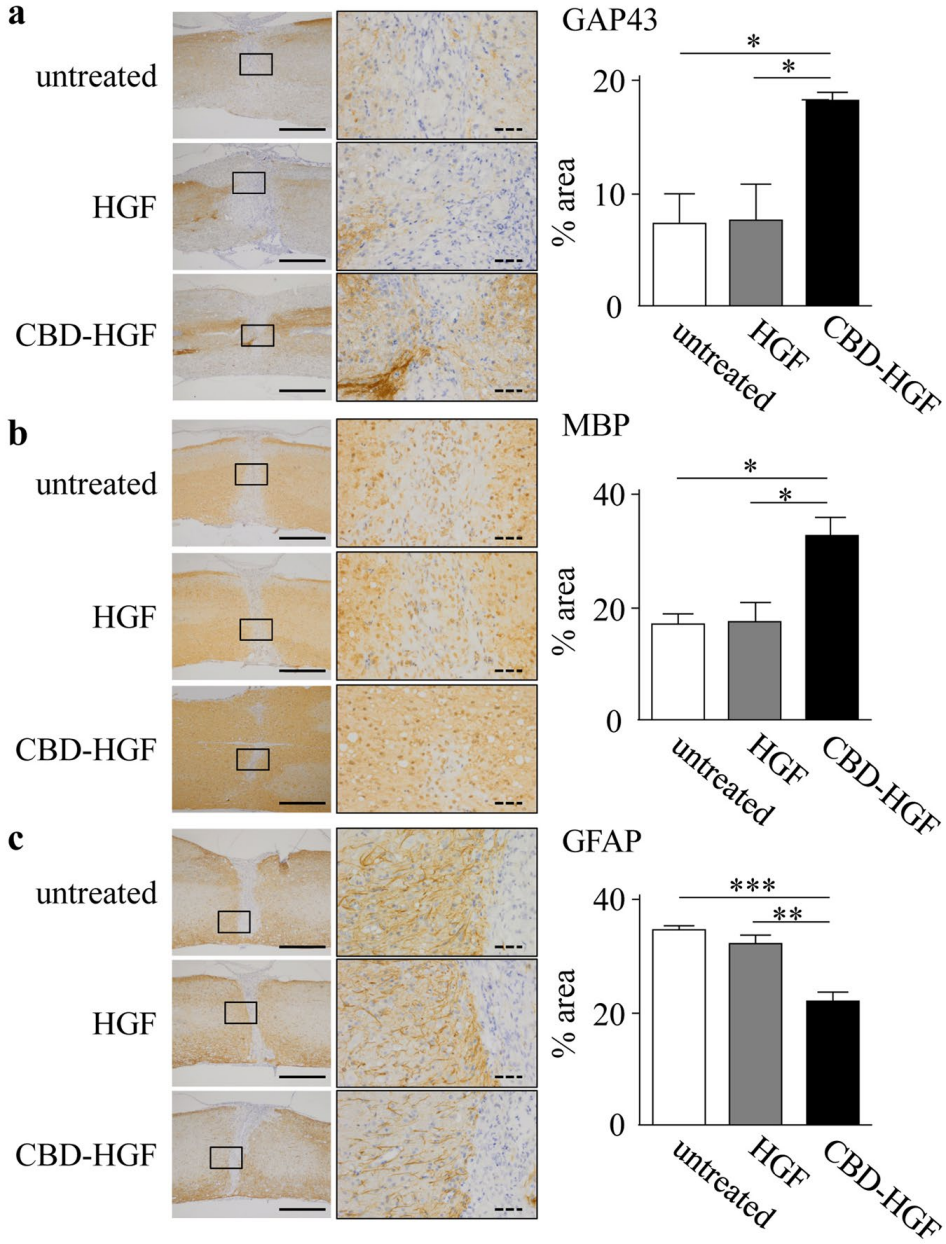
Axonal growth after spinal cord compression injury. Mice subjected to a compression injury were treated with a single administration of CBD-HGF or HGF. Untreated mice were used as controls (4 mice each per 3 groups). Immunohistochemistry for GAP43 (**a**), MBP (**b**) and GFAP (**c**) at the injury sites was performed 6 weeks post-injury. Left: Representative photographs from each group. The scale bar indicates 500 µm (solid line) and 50 µm (dotted line). Right: Immunopositive areas were measured under a microscope and percent area is shown. *p < 0.05, **p < 0.01, ***p < 0.001.

The prolonged binding of CBD-HGF to spinal cords may promote recovery from SCI more effectively than unmodified HGF. To address this, mice with a compression injury were treated with a single administration of HGF or CBD-HGF and locomotor functional recovery was assessed using the Basso mouse scale (BMS). Mice exhibited complete hind limb paralysis immediately after the injury (score 0), after which the mice recovered spontaneously (Fig. 2a). Although HGF treatment failed to improve the BMS score compared with the no treatment control, CBD-HGF significantly augmented the BMS score relative to the control (Fig. 2a). Motor-evoked potential (MEP) analysis was then performed as an electrophysiological assessment. The MEP amplitude in healthy mice was 650.2 ± 68.4 mV (data not shown). At 6 weeks after injury, the amplitudes in the untreated, HGF and CBD-HGF groups were 232.0 ± 45.9 mV, 229.0 ± 43.2 mV and 449.0 ± 61.5 mV, respectively (Fig. 2b). To investigate axonal growth, immunostaining for growth-associated protein 43 (GAP43; neuronal marker), myelin basic protein (MBP; oligodendrocyte/myelin marker) and glial fibrillary acidic protein (GFAP; astrocyte marker) was performed 6 weeks post-injury. The data in Fig. 3 demonstrated that both GAP43- and MBP-positive areas (Fig. 3a,b) in the CBD-HGF group were significantly larger than the other groups, while the GFAP-positive area (Fig. 3c) was significantly smaller, indicating scar formation was limited after CBD-HGF treatment. These results indicate that a single administration of CBD-HGF is more effective than unmodified HGF for the treatment of compression injury and is accompanied by neural regeneration.

### Augmented anti-inflammatory responses by CBD-HGF

**Figure 4:**
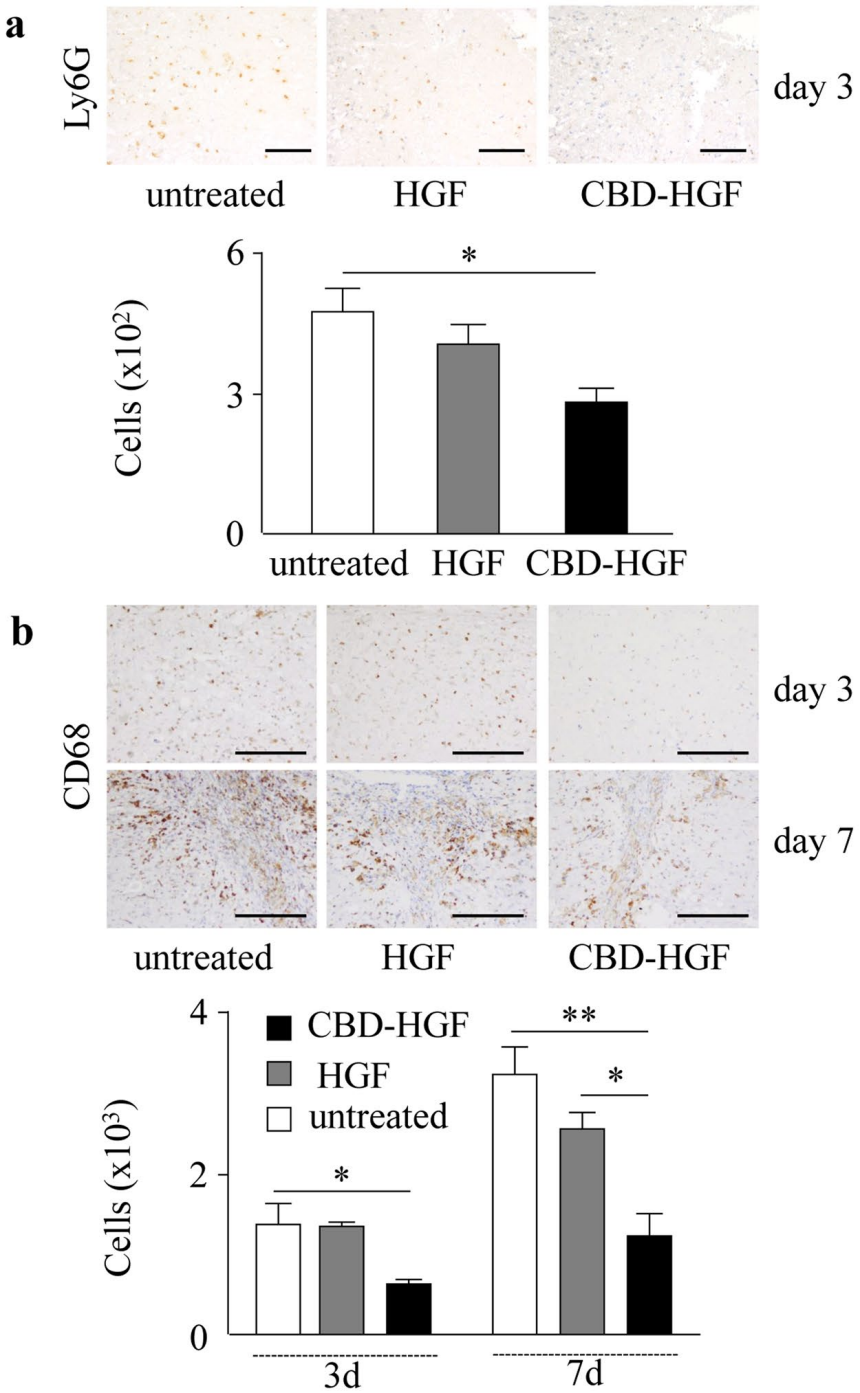
Leukocyte infiltration after spinal cord compression injury. Mice subjected to a compression injury were treated with a single administration of CBD-HGF or HGF. Untreated mice were used as controls (5 mice each per 3 groups). Immunohistochemistry for Ly6G (**a**) and CD68 (**b**) at the injury sites was performed 6 weeks post-injury. Upper: Representative photographs from each group. The scale bar indicates 100 µm (**a**) and 200 µm (**b**). Lower: The numbers of cells were counted under a microscope. *p < 0.05, **p < 0.01.

**Figure 5:**
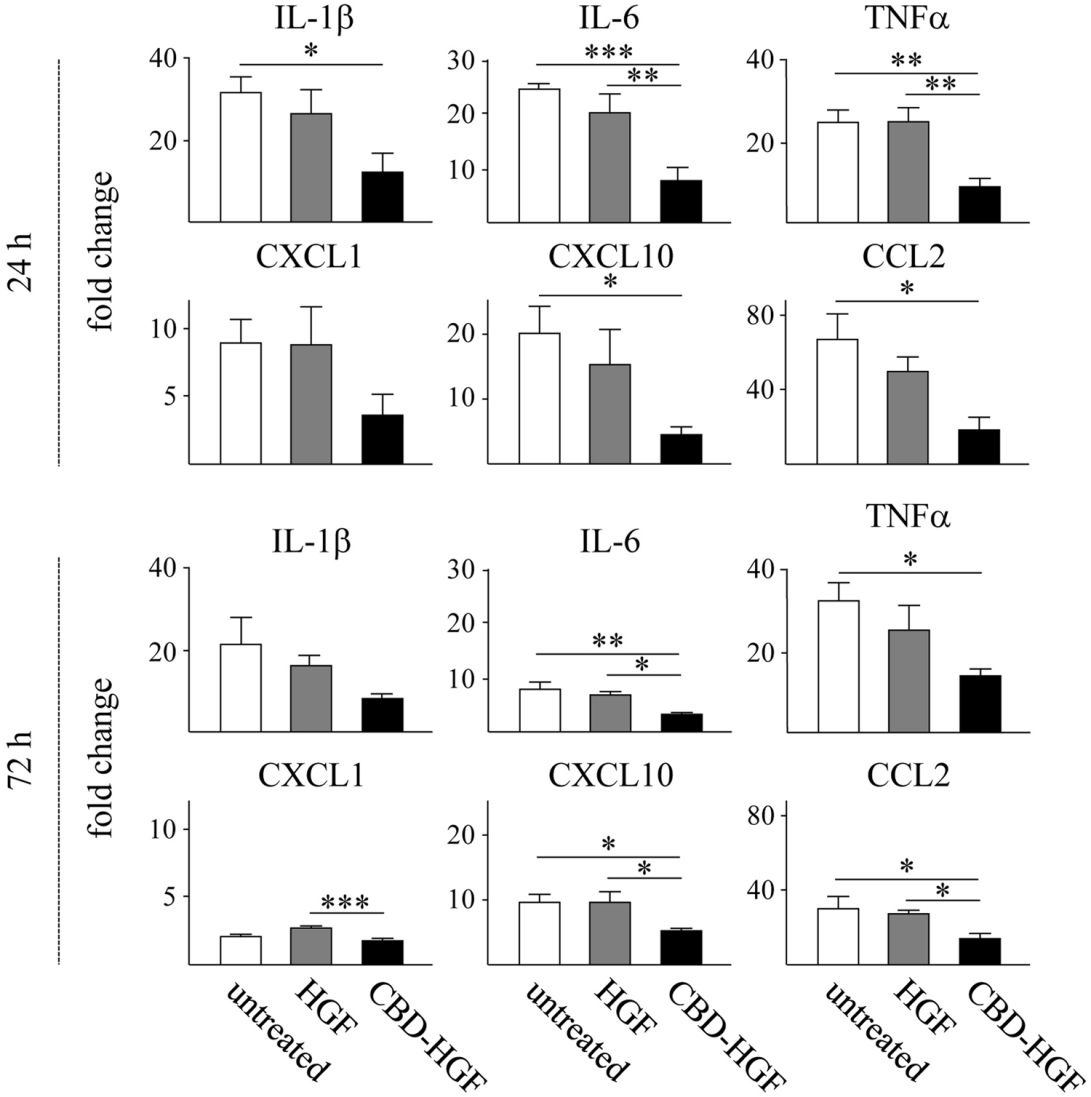
Cytokine and chemokine mRNA expression of after spinal cord compression injury. Mice subjected to a compression injury were treated with a single administration of CBD-HGF or HGF. Untreated mice were used as controls (4 mice each per 3 groups at each time point). Cytokine and chemokine mRNA expression at the injury sites was quantitated by qRT-PCR at 24 and 72 h after injury. The expression levels of each mRNA were normalised to *Gapdh* and the relative expression levels were compared with normal spinal cords. *p < 0.05, **p < 0.01, ***p < 0.01.

Spinal cord injury initiates a robust immune response represented by the production of cytokines and chemokines and a coordinated recruitment of leukocytes into the damaged site, which may result in further neural damage^19^. HGF is known to exert anti-inflammatory effects^20^. We confirmed that HGF suppressed the production of cytokines and chemokines from LPS-stimulated macrophages (Supplement data 1). We confirmed that HGF suppressed the production of cytokines and chemokines from LPS-stimulated macrophages (Supplemental data 1). We hypothesised that residual CBD-HGF could effectively induce an anti-inflammatory effect after a compression injury. As shown in Fig. 4, recruitment of neutrophils (Ly6G-positive, Fig. 4a) and macrophages (CD68-positive, Fig. 4b) was significantly reduced by CBD-HGF but not HGF treatment. T cell (CD3-positive) infiltration was barely detectable in all groups (data not shown). The expression of cytokines and chemokines in the spinal cord was then investigated. Compression injury induced the robust expression of several inflammatory cytokines and chemokines, all of which were significantly reduced by CBD-HGF but not by HGF (Fig. 5). Thus, CBD-HGF ameliorated inflammation, possibly through reducing cytokine/chemokine responses. Altogether, these data suggest that residual CBD-HGF promotes recovery from SCI more effectively than HGF through reduction of inflammatory responses after a compression injury.

### Treatment of complete transection injury by CBD-HGF-loaded gelatin-FA hydrogel

**Figure 6:**
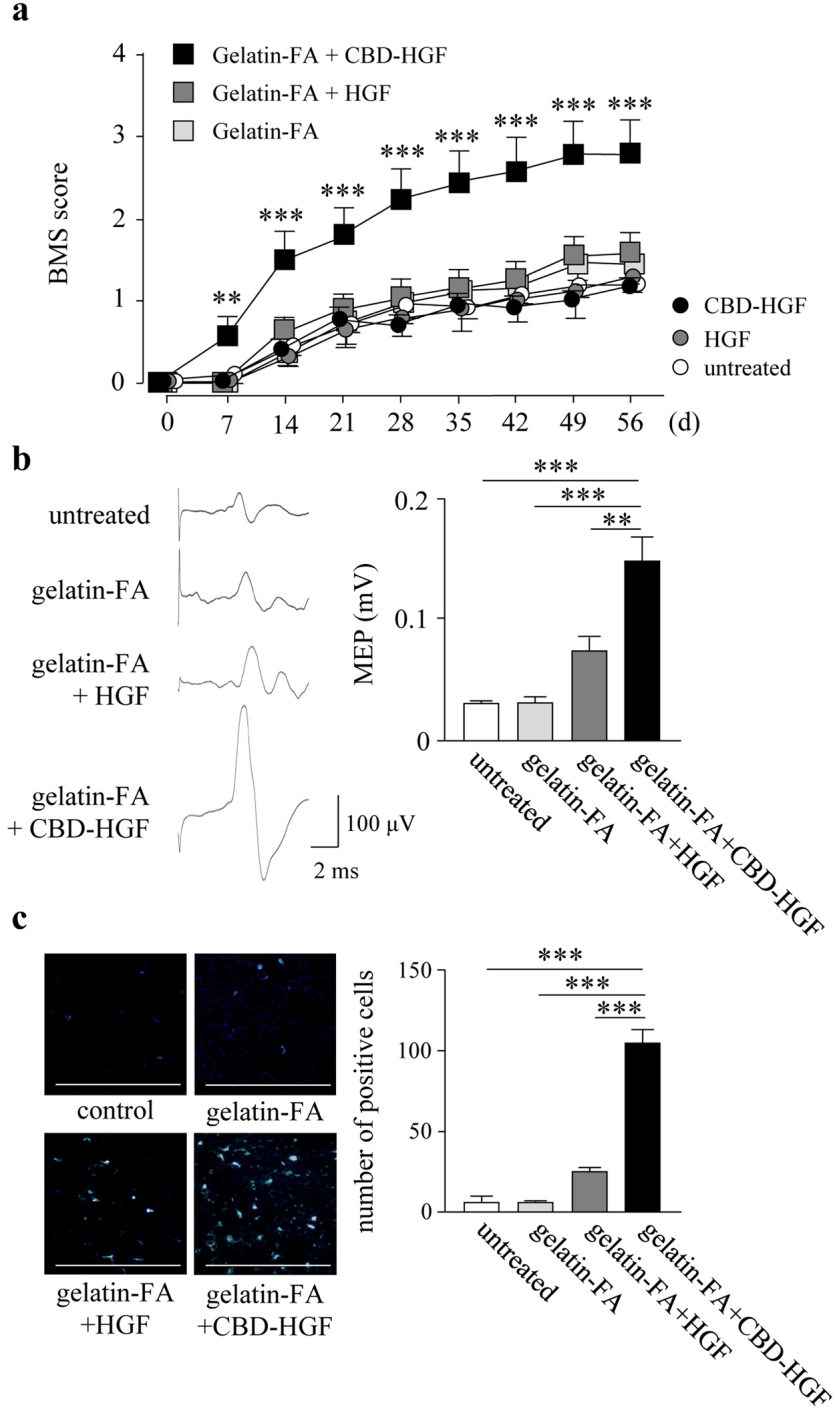
Treatment of spinal cord transection injury by the combination of CBD-HGF with or without a gelatin-FA hydrogel. Mice subjected to a transection injury were treated with a single injection of CBD-HGF or HGF alone, or a combination with gelatin-FA hydrogel (gelatin-FA hydrogel alone, gelatin-FA hydrogel + CBD-HGF or gelatin-FA hydrogel + HGF). Untreated mice were used as controls. (**a**) Motor function recovery was assessed at 8 weeks after injury using the BMS score (15 mice each per 6 groups). **p < 0.01, ***p < 0.01, versus the untreated group. (**b**) MEP analysis was performed as an electrophysiological assessment at 8 weeks after injury (5 mice each per 4 groups). Left: Representative data from each group. Right: Quantitative data from the 4 groups. **p < 0.01, ***p < 0.001. (**c**) Retrograde tracing was performed using Fluoro-Gold at 8 weeks after injury (5 mice each per 4 groups). Left: Representative data from each group. The scale bar indicates 500 µm. Right: Quantitative data from the 4 groups. ***p < 0.001.

Complete spinal cord transection mimics the severest clinical cases of SCI in humans^21^. The above results prompted us to apply CBD-HGF to the treatment of complete transection injury. Immediately after spinal cord transection, the mice exhibited complete hind limb paralysis (BMS score 0). A single administration of CBD-HGF failed to improve motor function, as compared to non-treated groups (Fig. 6a), leading to the idea that scaffold-based tissue engineering may be necessary, as scaffold provides an environment for cell attachment, proliferation and differentiation. For this, we employed a gelatin-FA hydrogel as a biocompatible scaffold. Mice subjected to complete spinal cord transection were treated with either HGF- or CBD-HGF-loaded gelatin-FA hydrogels, after which locomotor functional recovery was investigated. As shown in Fig. 6a, there were no significant differences in the BMS scores among non-treated, gelatin-FA and HGF-loaded gelatin-FA groups at each time point after transection. Interestingly, mice treated with CBD-HGF-loaded gelatin-FA showed significantly higher BMS scores than the other groups on day 7 and thereafter (Fig. 6a).

**Figure 7:**
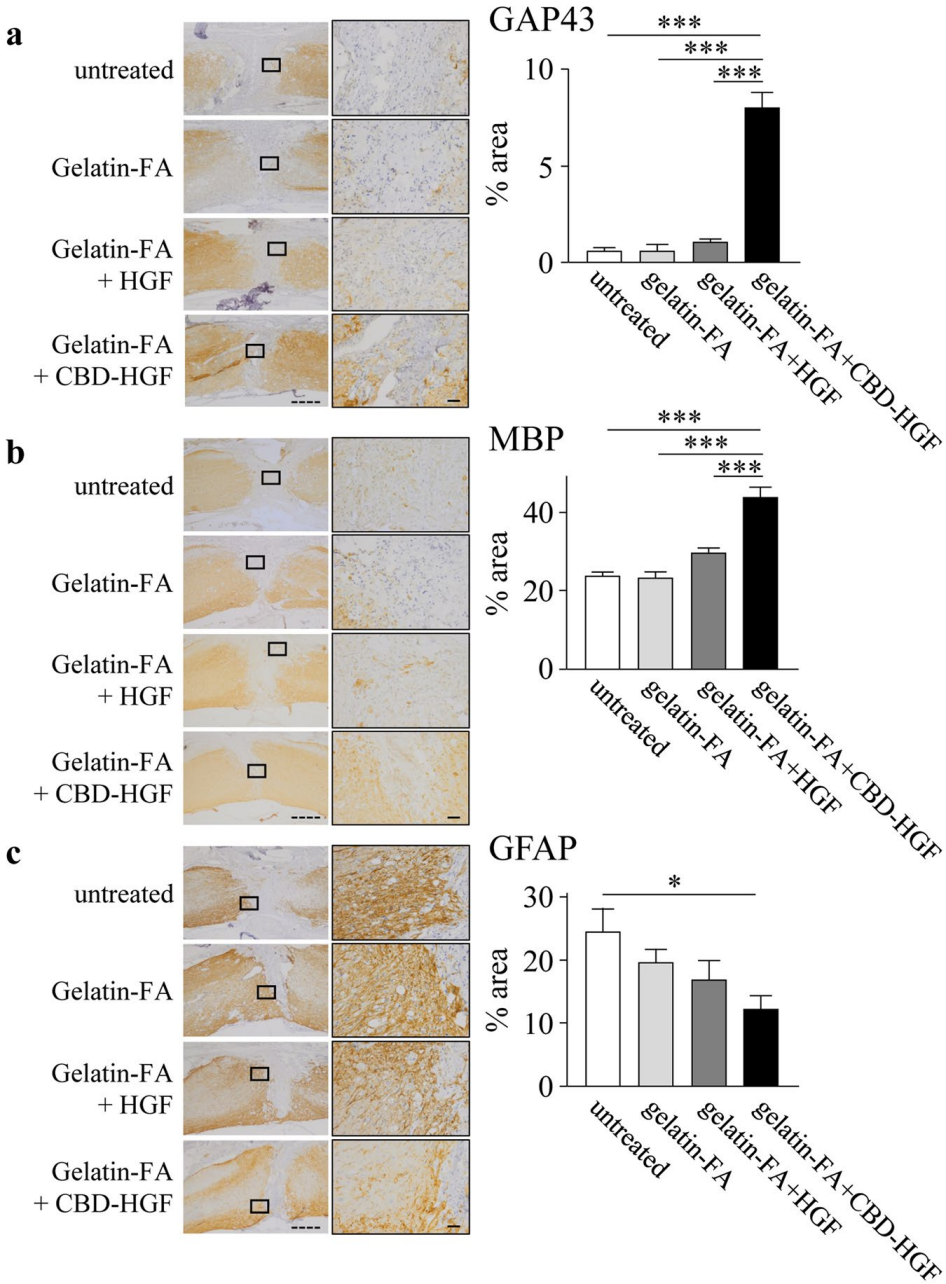
Axonal growth after spinal cord transection injury. Mice subjected to a transection injury were treated with gelatin-FA hydrogel alone, gelatin-FA hydrogel + CBD-HGF or gelatin-FA hydrogel + HGF. Untreated mice were used as controls (5 mice each per 4 groups). Immunohistochemistry for GAP43 (**a**), MBP (**b**) and GFAP (**c**) at the injury sites was performed 8 weeks post-injury. Left: Representative photographs from each group. The scale bar indicates 500 µm (dotted line) and 50 µm (solid line). Right: Immunopositive areas were measured under a microscope and percent area is shown. *p < 0.05, ***p < 0.001.

For further assessment, mice treated with gelatin-FA hydrogel groups were only examined, because therapeutic efficacy was not found without the scaffold. An electrophysiological assessment was performed by measurement of MEP amplitude. Before transection, the MEP amplitude was 650.2 ± 68.4 mV (data not shown). At 8 weeks after transection, the amplitude of MEPs in the untreated, gelatin-FA, HGF-loaded gelatin-FA and CBD-HGF-loaded gelatin-FA groups were 28.6 ± 2.4 mV, 30.1 ± 5.6 mV, 72.2 ± 10.7 mV and 146.0 ± 21.1 mV, respectively (Fig. 6b). The MEP amplitude of the gelatin-FA + CBD-HGF group was significantly higher than that of the other groups, including gelatin-FA + HGF. Next, we measured axonal regeneration by retrograde tracing of Fluoro-Gold (FG), which demonstrated that the number of FG-labelled neurons in the gelatin-FA + CBD-HGF group was significantly higher than in the other groups, including gelatin-FA + HGF (Fig. 6c). To further investigate axonal growth into the injury site, immunostaining for GAP43, MBP and GFAP was performed at 8 weeks after injury. As shown in Fig. 7, GAP43- and MBP-positive areas in the gelatin-FA + CBD-HGF group were significantly larger than the other groups (Fig. 7a,b). In contrast, the GFAP-positive area was significantly smaller (Fig. 7c). Altogether, these data suggest that gelatin-FA + CBD-HGF promotes recovery from complete transection injury more effectively than the other groups and that gelatin-FA appears to be a useful scaffold for severe SCI.

## Discussion

Growth factors have been applied to SCI as a potential therapeutic strategy; however, most clinical trials have failed because of lack of efficacy and/or unacceptable side effects^22^. To enhance the efficacy and at the same time reduce adverse effects, it will require an approach that limits the diffusion of factors into non-target tissues. The goal of tissue engineering approaches is to deliver a functional factor in sufficient quantities and to limit diffusion. Different approaches have been reported in animal models of SCI^22–24^; however, simpler methods to deliver growth factors to injury sites are desirable. In the present study, we applied CBD-HGF and gelatin-FA hydrogel to animal models of SCI. CBD-HGF was retained in the spinal cord and in a biocompatible gelatin-FA hydrogel much longer than native HGF. We demonstrated that CBD-HGF, but not HGF, improves motor function after compression SCI. Although CBD-HGF alone failed to promote recovery from complete transection SCI, CBD-HGF combined with gelatin-FA hydrogel was effective for the treatment of complete transection SCI.

SCI causes irreparable severe motor and sensory dysfunction through primary mechanical damage and secondary damage caused by the subsequent inflammatory responses^25^. Infiltrating leukocytes produce tissue damage by releasing lysosomal enzymes, proteases and reactive oxygen species^26,27^. To lessen the damage, it is important to control the secondary inflammatory response. HGF exerts anti-inflammatory effects through the disruption of NF-κB signalling and the subsequent expression of NF-κB-dependent proinflammatory mediators^20,28^. In this study, we demonstrated that infiltrating leukocytes such as neutrophils and macrophages were reduced by a single administration of CBD-HGF, an event that was associated with decreased levels of cytokines and chemokines at the injury site. To reduce inflammation, methylprednisolone has been administered to patients with acute SCI; however, pooled evidence from four randomised, controlled trials and 17 observational studies does not demonstrate a significant long-term benefit^29^. This indicates that an anti-inflammatory approach by itself is insufficient for the treatment of SCI. HGF is also known to have neurotropic activities^7,8,30^. HGF exhibits anti-apoptotic functions for neurons and oligodendrocytes, and inhibits glial scar formation^7–11^. In this study, we demonstrated that CBD-HGF significantly promoted axonal growth (increased GAP43- and MBP-positive areas), while limiting scar formation (reduced GFAP-positive area), as compared to HGF. Thus, in addition to its anti-inflammatory properties, retained CBD-HGF at the sites appears to recover SCI through neuroprotective activities.

Severe SCI often causes tissue damage leading to severe and permanent neurological deficits. Restoration of tissue continuity across tissue damage in severe injury lesions requires the implantation of scaffolds as oriented bridging structures that promote tissue remodelling^31,32^. The ideal scaffolds are biocompatible and can be designed to incorporate immobilised molecules, including growth factors, as drug delivery devices. CBD-HGF alone failed to improve recovery from complete transection injury; however, a combination strategy using a gelatin-FA hydrogel and CBD-HGF was effective for the treatment of transection injury. Because the binding capacity of CBD-HGF to hydrogels was equal to tissue, gelatin-FA hydrogels appear to provide an optimal environment in tissue regeneration. Electrophysiological and retrograde tracing outcomes and immunohistochemical studies demonstrated that the combination strategy provided structural guidance for axonal regeneration and prevented the formation of scar tissue. Thus, CBD-HGF retained in the scaffolds possibly promoted recovery from complete SCI. Gelatin is a biocompatible, biodegradable and manoeuvrable material with no harmful effects. Photo-cross-linkable gelatin can be used to form scaffolds *in situ* from injectable solutions and visible light^18,33^. Treatment strategies using photo-cross-linkable gelatin-FA hydrogel with CBD-HGF may be beneficial for the treatment of serious spinal cord damage after SCI.

There are several concerns that were not addressed in this study. We believe the anti-inflammatory properties of CBD-HGF are important in the functional recovery from compression injury; however, the precise mechanism(s) and the degree of contribution remain unclear. It will be important to determine whether CBD-HGF combined with a gelatin-FA hydrogel could further enhance the functional recovery from compression injury. In the transection model, how gelatin-FA hydrogel supports tissue repair and when gelatin-FA hydrogel is absorbed and replaced by regenerated tissue remain unknown. Although CBD-HGF in combination with a gelatin-FA hydrogel was effective for the treatment of transection injury, the functional recovery was not fully satisfactory. Recent animal studies have shown that hydrogels and stem cell-based therapies enhance functional recovery after SCI^31,34,35^. Very recently, Takano *et al*. has demonstrated that HGF plays a key role in the enhanced functional recovery after neural stem cell transplantation observed in aged mice with SCI^36^. An attractive question is whether a combination strategy with stem cells and CBD-HGF in gelatin-FA hydrogel could generate an additive or synergistic effect to increase the recovery from complete SCI. Further studies are necessary to address these possibilities.

In conclusion, we employed CBD-HGF and photo-cross-linkable gelatin-FA as a tissue-engineering approach. Our engineered CBD-HGF promoted *in vivo* axonal regeneration even by a single injection at the targeted site. They are many ways to enhance the release of HGF using continuous injection, virus vector or HGF overexpressing mesenchymal stem cells^9,10,12^. We believe that our fusion protein is better than others as CBD-HGF can localize longer at the designated site, resulting in effective neural recovery by a single low dose administration. In combination with gelatin-FA scaffold, CBD-HGF improved recovery from severe complete transection injury. Gelatin-FA may provide an optimum environment for neural regeneration and synergistic effects with CBD-HGF. Thus, CBD-HGF combined with hydrogel scaffold may be promising for the treatment of serious SCI.

## Methods

### Reagents

HGF fused to an additional collagen-binding domain derived from fibronectin (CBD-HGF) was prepared by a baculovirus expression system as described^13^. Control human HGF was purchased from R&D Systems (Minneapolis, MN, USA). CBD-HGF and HGF were labelled with HiLyte Fluor 555 (Dojindo Molecular Technologies, Inc., Kumamoto, Japan)^37^. Porcine skin gelatin (G2500), furfurylamine (FA) and Rose Bengal (RB) were purchased from Sigma-Aldrich (St. Louis, MO, USA). Gelatin-FA was prepared as described^18^. Anti-GAP43, anti-GFAP, anti-MBP and anti-CD68 were purchased from Abcam (Cambridge, UK). Anti-Ly6G and anti-CD3 antibodies were from BioLegend (San Diego, CA).

### Mice

Female C57Bl/6 mice (8–9 weeks old) were obtained from Charles River Laboratories (Kanagawa, Japan). The mice were housed in a temperature-controlled environment with a 12 h light/12 h dark cycle and allowed free access to water and food. The animal care and use committee at the Okayama University approved all experimental protocols for this study. All experiments were performed in accordance with relevant guidelines and regulations.

### *In vivo* imaging of CBD-HGF

Mice were anesthetised by intraperitoneal injection of ketamine (100 mg/kg). A complete laminectomy was performed at T9 and fluorescently labelled CBD-HGF or HGF was intrathecally injected (2 µl) into the spinal cord. In the case of the combination strategy with the gelatin scaffold, a complete laminectomy was performed at T8-T9. A 3 mm-diameter hole was made in the dura mater with a microscalpel and 15 µl of gelatin solution (15% gelatin-FA with 0.05% RB in PBS) was applied and gelled by irradiation with visible light (Luminar Ace LA-HDF158A, Hayashi Watch-works Co., Tokyo, Japan) for 1 min. We showed that gelatin-FA hydrogels had higher elasticity and flexibility compared to existing gelatin hydrogels^18^. The fascia and the skin were sutured separately. All mice were anesthetised, then euthanized by transcardial perfusion with 4% PFA at 1, 3 or 7 days after substance administration. The spinal cords and surrounding tissues were removed, embedded in optimal cutting temperature compound, frozen and sectioned in the sagittal plane at 12 µm on a cryostat. The tissue sections were evaluated by fluorescence microscopy.

### CBD-HGF in gelatin-FA hydrogel *in vitro*

A total of 60 µl of gelatin solution (15% gelatin-FA and 0.05% RB in PBS) containing CBD-HGF or HGF (each 100 ng) was added to a 96-well enzyme-linked immunosorbent assay (ELISA) plate and then gelled by irradiation with visible light for 1 min. The samples were incubated with 200 µl of PBS at 37 °C and remaining hydrogels were harvested at 1, 3, 5 and 7 d. The hydrogels were mechanically disrupted after adding 140 µl of PBS. Concentrations of HGF in the solutions were measured by ELISA (R&D Systems, Minneapolis, MN).

### Spinal cord injury models

We employed two models of SCI: a compression and a transection model. For the compression model, mice were subjected to laminectomy at T9 and the spinal cord was compressed with a vascular clip (10 g force, 1 min)^38^ under anaesthesia. Immediately after the injury, mice were intrathecally injected with 2 µl of either PBS, CBD-HGF or HGF (each 1 µg). The fascia and the skin were then sutured separately. For the transection model, the spinal cord and nearby tissues were completely transected with a microscalpel after laminectomy at T9^39^. The injury site was implanted with 15 µl of gelatin solution (15% gelatin-FA and 0.05% RB in PBS), gelatin solution with unmodified HGF (1 µg) or gelatin solution with CBD-HGF (1 µg), and gelled by irradiation with visible light for 1 min. Untreated injured spinal cord tissue was used as a control. The fascia and the skin were sutured separately. At appropriate intervals after the treatment, mice were euthanized and the spinal cords were resected. For immunohistochemistry, tissues were fixed in 4% paraformaldehyde and embedded in paraffin. For mRNA expression, samples were immediately frozen in liquid nitrogen and stored at −80 °C until use.

### Assessment of motor function recovery

Motor recovery after SCI was evaluated by the Basso mouse scale (BMS)^40^. The scale ranges from 0 (no ankle movement) to 9 (complete functional recovery). BMS scores were recorded at 7 days and then every other week after SCI by two independent examiners who were blinded to the experimental conditions. Hind-limb motion was used to assess coordinated movement and stepping. When differences in the BMS score between the right and left hind limbs were observed, the average of the two scores was used. All experiments were performed in accordance with relevant guidelines and regulations.

### Electrophysiological evaluation

Electrophysiological analyses were performed at 8 weeks after SCI, as described^41,42^. Under anaesthesia, motor-evoked potentials (MEPs) were elicited by stimulation of the spinal cord at the occipito-cervical interspace. Recording electrodes in the hind-limb were placed on the muscle belly (recording) and the distal tendon of the muscle (reference). The ground electrode was placed subcutaneously between the coil and the recording electrodes. Stimulus intensity was adjusted to 10–20% above the voltage at which the maximum amplitude of the initial peak of the evoked response was observed. The amplitude (µV) was measured from the initiation point of the first wave to its highest point.

### Retrograde tracing

To trace neural connectivity of the spinal cord after transection injury, retrograde tracing was carried out^43^. At 8 weeks after transection of the spinal cord at T9, mice were subjected to another laminectomy at T12, followed by injection of 0.5 µl of 4% retrograde tracer Fluoro-Gold (FG; Fluorochrome, LLC, Denver, CO) into the spinal cord. Three days after FG injection, the mice were transcardially perfused with 4% PFA. Brainstems were dissected and placed in 30% sucrose for cryopreservation. Serial frozen coronal sections were cut, and numbers of FG-labelled neurons in the red nucleus were counted under fluorescence microscopy.

### Quantitative real-time PCR (qRT-PCR)

Samples were homogenized in Lysis buffer (Quickgene, Fujifilm, Tokyo, Japan), and total RNA was isolated according to the manufacturer’s instructions. Extracted RNA was reverse-transcribed using ReverTra Ace qPCR RT kit (Toyobo Life Science, Osaka, Japan) and qRT-PCR was performed using the TaqMan 7500 sequence detection system (Applied Biosystems, Foster city, CA). The expression of *IL-1b*, *IL-6*, *TNFa*, *CXCL1*, *CXCL10*, *CCL2* and *Gapdh* genes was analysed by Taqman gene expression assays (Applied Biosystems). The expression level of each gene was normalized to that of the *Gapdh* gene and presented as fold change over the expression of the control gene.

### Immunohistochemistry

Immunostaining was carried out using the Histofine Simple Stain MAX-PO kit (Nichirei Biosciences Inc., Tokyo, Japan), according to the manufacturer’s instructions. In brief, sections for microscopy (4 µm) were treated with 0.3% H_2_O_2_ in methanol and then incubated with primary antibodies overnight at 4 °C. Sections were rinsed and incubated with peroxidase-labelled polymer at room temperature for 30 min. As a chromogen, diaminobenzidine (DAKO, Carpinteria, CA) was used. Stained cells were counted using microscopy and ImageJ software.

### Statistics

Data were analysed using StatMate IV software version 4.01 (Advanced Technology for Medicine and Science, Tokyo, Japan). All data were expressed as mean ± SEM. An unpaired two-tailed Student’s t-test was used to evaluate the differences between groups for the *in vitro* collagen binding analysis. One-way ANOVA followed by the Tukey-Kramer test for multiple comparisons was used for immunohistochemistry, electrophysiological evaluation and retrograde tracing. Repeated-measures two-way ANOVA followed by the Tukey-Kramer test was used for the BMS analysis. For all analyses, *p*-values smaller than 0.05 were considered significant.

## Electronic supplementary material

Supplementary Information
