# Supplementary material for: Collagen-Binding Hepatocyte Growth Factor (HGF) alone or with a Gelatin- furfurylamine Hydrogel Enhances Functional Recovery in Mice after Spinal Cord Injury
Source: Sci Rep. 2018 Jan 17;8:917. doi: 10.1038/s41598-018-19316-y (PMC5772669; doi:10.1038/s41598-018-19316-y)
Supplement: Supplementary file 1 — Supplementary Information [file 41598_2018_19316_MOESM1_ESM.pdf]

Collagen-Binding Hepatocyte Growth Factor (HGF) alone or with a Gelatin- furfurylamine Hydrogel Enhances Functional Recovery in Mice after Spinal Cord Injury

Kentaro Yamane, Tetsuro Mazaki, Yasuyuki Shiozaki, Aki Yoshida, Kensuke Shinohara, Mariko Nakamura, Yasuhiro Yoshida, Di Zhou, Takashi Kitajima, Masato Tanaka, Yoshihiro Ito, Toshifumi Ozaki, and Akihiro Matsukawa

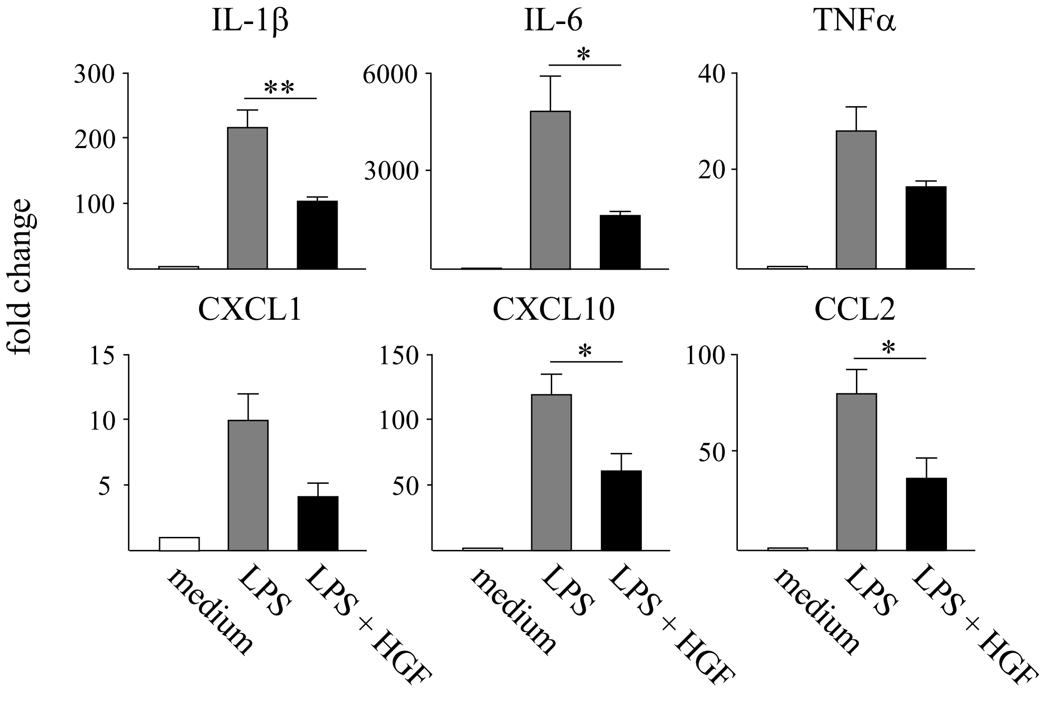

Supplement data 1

Bone marrow (BM) was isolated from healthy mice and BM cells were cultured in L929 cell conditioned medium. Six days later, BM-derived macrophages (BMDMs) were harvested, seeded into 24-well plates (1×10^4 cells/well) and cultured overnight at 37°C. BMDMs were then stimulated with 100 ng/mL LPS for 6 h in the presence or absence of HGF (50 ng/ml) and mRNA expression of cytokines and chemokines was quantitated by qRT-PCR (n = 3 each); *$p < 0.05$, **$p < 0.01$.
